# Multilevel Conduction Abnormalities: Hickam’s Dictum or Occam’s Razor?

**DOI:** 10.19102/icrm.2022.130603

**Published:** 2022-06-15

**Authors:** Sharath K. Kumar, Sheldon M. Singh

**Affiliations:** ^1^Schulich Heart Program, Sunnybrook Health Sciences Centre, Toronto, Ontario, Canada; ^2^Faculty of Medicine, University of Toronto, Toronto, Ontario, Canada

**Keywords:** Atrioventricular block, concealed His-bundle extrasystole, electrocardiogram

## Abstract

A 62-year-old woman with a spinal cord injury and no prior cardiac disease presented with an abnormal electrocardiogram. A systematic evaluation of the electrocardiogram suggested a diagnosis of concealed His extrasystole. This case report features an interesting phenomenon of pseudo-atrioventricular block due to concealed junctional discharges.

## Introduction

Type 2 second-degree atrioventricular (AV) block is due to an infra-nodal conduction disturbance and is an indication for pacemaker therapy. Concealed discharges from the AV junction may mimic type 2 second-degree AV block by interfering with antegrade conduction. This entity is fundamentally a disturbance of impulse formation with premature discharges from the AV junction interfering with AV conduction. This electrophysiologic entity needs to be distinguished from “true” AV block and has different therapeutic implications. The present report illustrates this phenomenon.

## Case presentation

**Figure 1:**
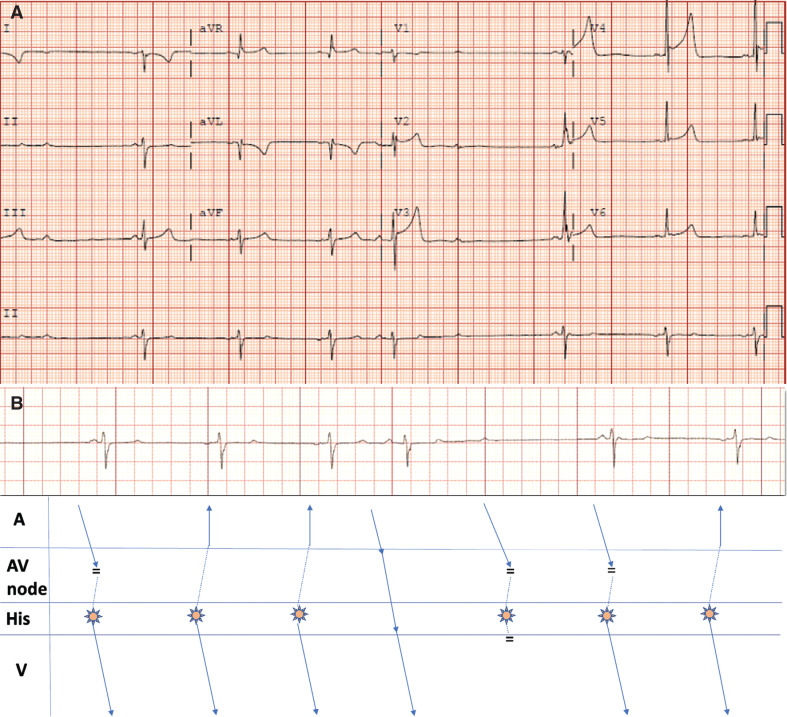
Concealed His-bundle extrasystoles. **A:** Twelve-lead electrocardiogram highlighting an abnormal rhythm. **B:** Rhythm strip with accompanying ladder diagram. Starburst corresponds to the concealed His extrasystole. The black bard represents collision. *Abbreviation:* AV, atrioventricular.

A 62-year-old woman with a spinal cord injury was evaluated for an abnormal electrocardiogram (ECG) **(Figure 1A)**. She had no prior history of heart disease, all electrolytes were normal, and she was not on medications known to cause bradyarrhythmia. The echocardiogram revealed normal biventricular function. The lead II rhythm strip **(Figure 1B)** demonstrates multiple findings, including (1) 2 P-wave morphologies, (2) non-conducted P-waves, and (3) P–R-interval variation without QRS pre-excitation. We believe these findings can all be explained by a single phenomenon—concealed His-bundle extrasystoles.

In **Figure 1**, the first P-wave that is upright in the inferior leads is a sinus P-wave that occurs almost simultaneously with a His-bundle extrasystole. This extrasystole subsequently conducts to the ventricle with a normal QRS without the need for AV nodal conduction, resulting in a short P–R interval. Rhythmic ectopics from the His bundle continue with 2 subsequent inverted P-waves and narrow QRS intervals with a short P–R interval due to simultaneous retrograde atrial conduction and anterograde His–Purkinje activation. The fourth beat reveals an upright P-wave with a P–R interval of 190 ms and represents a normally conducted sinus P-wave pre-empting the extrasystole. The subsequent sinus impulse does not propagate to the ventricle due to a concealed His ectopic. We posit that anterograde ventricular conduction fails to occur due to ventricular activation occurring during its effective refractory period. The final 2 beats are similar to the initial 2 beats of the tracing. Of note, the ECG also shows left- and right-arm lead reversals.

Given the transient nature of this ectopy associated with spinal cord injury, an electrophysiological study was not performed. The patient was managed with β-blockers and the episodes subsided.

## Discussion

Pseudo-AV block due to concealed ectopic beats arising from the His bundle should be considered in the differential diagnosis of unexplained AV block, especially when the QRS complex is narrow. P-wave variability may also be evident due to retrograde atrial activation. Concealed conduction is defined as an impulse that manifests itself in the subsequent beat.^1^ In this situation, AV block is a manifestation of concealed conduction of extrasystoles arising in the His-bundle region. It has to be realized that AV block in this case is due to a disorder of impulse formation rather than due to a conduction problem; hence, it is known as pseudo-AV block.^2^

The mechanism of this phenomenon is automaticity in the His region in an intermittent manner or, rarely, in a parasystolic fashion. This extrasystole may manifest in the following different ways: (1) antegrade conduction with or without retrograde penetration; (2) aberrantly conducted antegrade impulse with or without retrograde atrial activation; (3) retrograde atrial conduction without antegrade conduction; or (4) concealed conduction, where the impulse is not transmitted both antegrade and retrograde but manifests itself in the subsequent beat. When such a beat encounters an atrial impulse, it blocks antegrade conduction, thereby manifesting as AV block.^3^ Concealed His extrasystoles as a cause of AV block can be suspected when there are junctional beats with episodes of type I or type II AV block. Additional features that are pointers to this phenomenon are sudden unexpected variations in the P–R interval during 1:1 conduction and atrial fusion beats.^2,3^ The present case illustrates these diagnostic features with junctional rhythm and blocked P-waves.

A diagnostic electrophysiological study may not be required because His extrasystoles may be fortuitous. Recognizing this phenomenon is important as pacing therapy is not needed in most cases unless there is clear evidence of a compromised His–Purkinje system. If persistent and symptomatic, interventions that suppress ectopy, such as β-blockers or ablation, may be considered.^4^ Drugs such as lidocaine and procainamide have also been shown to be useful.^5^

In conclusion, His-bundle extrasystoles should be considered in the presence of apparent multi-level conduction abnormalities. A careful deductive analysis of the ECG is helpful in arriving at a diagnosis.
